# Effects of *Trichoderma harzianum* on Photosynthetic Characteristics and Fruit Quality of Tomato Plants

**DOI:** 10.3390/ijms22136961

**Published:** 2021-06-28

**Authors:** Igor D. Vukelić, Ljiljana T. Prokić, Gordana M. Racić, Mirjana B. Pešić, Mirjana M. Bojović, Edyta M. Sierka, Hazem M. Kalaji, Dejana M. Panković

**Affiliations:** 1 Faculty of Ecological Agriculture, Educons University, Vojvode Putnika 87, 21208 Sremska Kamenica, Serbia; igor.vukelic@educons.edu.rs (I.D.V.); gordana.racic@educons.edu.rs (G.M.R.); mirjana.bojovic@educons.edu.rs (M.M.B.); 2 Faculty of Agriculture, University of Belgrade, Nemanjina 6, 11080 Belgrade, Serbia; ljprokic@agrif.bg.ac.rs (L.T.P.); mpesic@agrif.bg.ac.rs (M.B.P.); 3 Faculty of Natural Sciences, Institute of Biology, Biotechnology and Environmental Protection, University of Silesia in Katowice, 28 Jagiellonska, 40-032 Katowice, Poland; edyta.sierka@us.edu.pl; 4 Department of Plant Physiology, Institute of Biology, Warsaw University of Life Sciences—SGGW, 02-787 Warsaw, Poland; hazem@kalaji.pl; 5 Institute of Technology and Life Sciences, National Research Institute, Falenty, Al. Hrabska 3, 05-090 Raszyn, Poland

**Keywords:** organic agriculture, polytunnel, *Solanum lycopersicum* L., symbiotic endophyte

## Abstract

The beneficial role of fungi from the *Trichoderma* genus and its secondary metabolites in promoting plant growth, uptake and use efficiency of macronutrients and oligo/micro-nutrients, activation of plant secondary metabolism and plant protection from diseases makes it interesting for application in environmentally friendly agriculture. However, the literature data on the effect of *Trichoderma* inoculation on tomato fruit quality is scarce. Commercially used tomato cultivars were chosen in combination with indigenous *Trichodrema* species previously characterized on molecular and biochemical level, to investigate the effect of *Trichoderma* on photosynthetic characteristics and fruit quality of plants grown in organic system of production. Examined cultivars differed in the majority of examined parameters. Response of cultivar Gružanski zlatni to *Trichoderma* application was more significant. As a consequence of increased epidermal flavonols and decreased chlorophyll, the nitrogen balance index in leaves has decreased, indicating a shift from primary to secondary metabolism. The quality of its fruit was altered in the sense of increased total flavonoids content, decreased starch, increased Bioaccumulation Index (BI) for Fe and Cr, and decreased BI for heavy metals Ni and Pb. Higher expression of swolenin gene in tomato roots of more responsive tomato cultivar indicates better root colonization, which correlates with observed positive effects of *Trichodrema*.

## 1. Introduction

One of the main goals of modern sustainable agriculture is to support raising demands for food and energy of a growing world population but at the same time to maintain soil health and fertility. Soil microorganisms, soil type, agricultural management and plant genotype play crucial roles in determination of soil functioning and fertility [1].

With a production of about 180 million tons in 2017 (FAOSTAT), tomato (*Solanum lycopersicum* L.) is one of the most important cultivated vegetable in the world. Tomato fruit is a source of vitamins, carotenoids, carbohydrates, phenolic compounds and nutrients which are of vital role in human diet. Among many phenolic compounds in tomato fruit, flavonoids are contributing to its anti-oxidative, anti-cancer, anti-diabetes and cardiovascular protective effects [2]. Carbohydrate content in tomato fruit is considered to be strongly related to fruit yield and quality, as it has important role in fruit growth, composition and ripening [3]. Containing only about 4% of carbohydrate, it is considered as non-starchy vegetable and advised to be used in weight-loss diets [4].

Many factors affect tomato fruit quality: plant nutrients, climatic factors, physical and chemical soil properties and fertilization [5,6]. The Bioaccumulation Index (BI) is often used to investigate the plant efficiency for uptake of elements from the soil and accumulate it in the edible part of the plant [7]. In general, plant symbionts are affecting the uptake of nutrients from the soil [8]; however, their influence on plant metal uptake are still poorly understood [9]. *Trichoderma* fungi are rhizosphere microorganisms often in symbiotic interaction with plants. The literature data on the effect of *Trichoderma* inoculation to the plant uptake of nutrients are not consistent. In some cases, the uptake was increased [10] and in others decreased [11]. The plant uptake of nutrients as influenced by *Trichoderma* is considered to be a key factor for many positive effects of this fungi to plant status, even more so nutrient availability in soils influences the biocontrol efficacy of *Trichoderma* species [12].

The beneficial properties of *Trichoderma* and its secondary metabolites on plants concerning improved plant growth parameters, uptake of macronutrients and oligo/micro-nutrients, water use efficiency, photosynthesis, activation of plant secondary metabolism and accumulation of polyphenols have been observed [13,14]. Increased hormone accumulation, altered sugar partitioning, enhanced photosynthetic efficiency and growth promotion of tomato plants were induced by specific strain of *T. harzianum* [15]. Improved photosynthetic capabilities of different plant species induced by various endophytic strains of *Trichoderma* due to the increase of photosynthetic pigments or gene expressions regulating chlorophyll biosynthesis, light harvesting complex proteins or Calvin cycle components were documented in more than twenty papers [16]. The upregulation of plant genes or proteins providing optimized internal redox environment in response to abiotic of biotic stresses when roots are colonized by *Trichoderma* strains was even more significant. However, information about its effect on tomato fruit quality in organic cultivation is scarce. To our knowledge, only two studies on the effect of different *Trichoderma* species on tomato fruit quality were conducted [13,17]. In the first one, *T. longibrachiatum* significantly promoted the growth and yield of tomato, obtaining fruits with higher titratable acidity and lower total soluble solids (TSS) content [17]. In the second one the total yield, contents of lycopene and some amino acids increased in the plum tomato fruits after the application of *T. harzianum*, which was discussed in terms of improved nitrogen metabolism [13].

The recognition between *Trichoderma* and its host during symbiotic associations is mediated by effectors [18,19,20]. *Trichoderma* colonizes root surfaces and penetrates to the first or second layers of cells, which is achieved by secretion of cellulolytic and proteolytic enzymes, for example, swolenin that can disrupt the crystalline cellulose structure of plant cell walls [21,22].

The aim of this paper was to study the long-term effects of *T. harzianum* application on two tomato cultivars (Narvik and Gružanski zlatni) grown in field conditions under polytunnel. For that purpose, we have selected strain *T. harzianum* SMZC 22660, as it previously exhibited positive effects on the growth of tomato plants, content of epidermal flavonols and decreased the uptake of toxic elements (Cd, Ni and Cr) in a growth chamber experiment [23]. In addition, all examined extracellular enzyme activities of this strain were high, in particular plant cell wall-degrading enzymes which are important for good root colonization. The effects on photosynthetic characteristics were examined by non-destructive measurements on plant leaves. Moreover, fruit was examined for morphological characteristics, and in order to evaluate fruit quality, total phenolic, total flavonoid, soluble sugars and starch content, and uptake efficiency of nutrients and heavy metals were measured.

## 2. Results

Prior to application, *T. harzianum* SMZC 22660 was characterized by measurement of extracellular enzyme activities (Table 1). High activities of *N*-acetyl-β-glucosaminidase, acid phosphatase and naphtol-AS-BI-phosphohydrolase were observed (classified according to the color scale), while low activities were determined for alkaline phosphatase, esterase C4, esterase lipase C8. The suspension of *T. harzianum* spores (8 × 10^6^ CFU/mL) was applied in the root zone of tomato plants at the beginning of flowering phase and the measurement were done 60 days after application. The plants that were grown in the polytunnel were optimally irrigated by the drip system as confirmed by the SWC and RWC of the leaves (Table 1).

**Figure 1:**
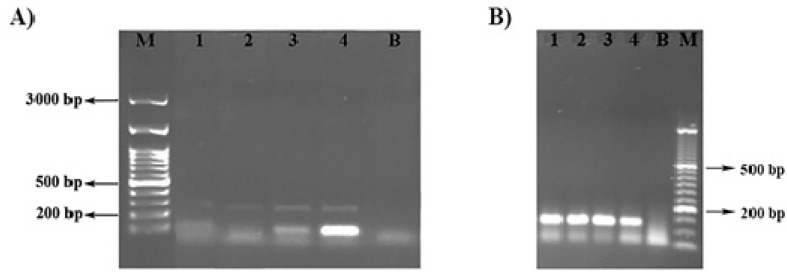
Expression of the swolenin gene (**A**) and β-actin reference gene (**B**) in the roots of tomato variety Narvik (1, 2) and Gružanski zlatni (3, 4). B refers to blank and M referes to the 100 bp DNA Ladder H3 RTU 50 μg/500 μL (**A**) and 50 bp DNA Ladder MWD50 50 μg/500 μL (**B**).

The colonization of the roots of two examined tomato varieties was checked by the expression analysis of swolenin, one of the *Trichoderma* effector proteins. The expression was lower in the roots of Narvik (lanes 1 and 2 represent two replicate bulk samples) than in Gružanski zlatni (lanes 3 and 4 represent two replicate bulk samples) (Figure 1).

Several morpho-physiological parameters of plants and fruits as well as some fruit quality parameters were measured.

The content of chlorophyll was higher in cultivar Gružanski zlatni in general. However, *Trichoderma* treated plants exhibited 13% lower chlorophyll content, which was not the case in cultivar Narvik. The response of the content of flavonols was opposite in examined cultivars: in Narvik it decreased by 12%, while in Gružanski zlatni it increased by 25%, in response to *Trichoderma* treatment. Consequently, NBI has decreased by 21% with treatment in Gružanski zlatni. Generally, NBI was lower in Narvik than in Gružanski zlatni. In both cultivars the content of anthocyanins was low and did not change after *Trichoderma* treatment.

According to most of the examined morphological parameters of tomato plants and fruits, there were no significant differences neither between cultivars, nor between *Trichoderma* treatments and controls (Table 2). The most significant difference between the examined cultivars was the number of fruits, almost doubled in cultivar Gružanski zlatni, but it did not respond to *Trichoderma* treatment.

Content of total polyphenols in the fruit significantly decreased to the similar extent in both investigated genotypes with addition of *Trichoderma*, 13.7% for Narvik and 14.5% for Gružanski Zlatni (Table 3). Similarly, significant decrease (26%) of flavonoid content was observed when *Trichoderma* was applied in genotype Narvik; however, flavonoids increased by 48.9% in Gružanski zlatni. The decrease of starch content (37%) after *Trichoderma* treatment was observed in the same cultivar. Significantly lower content of soluble sugars was observed in tomato fruits of cultivar Narvik in comparison to Gružanski zlatni; however, there were no significant changes with *Trichoderma* treatment.

Nitrogen content in Cultivar Narvik was 1.28 ± 0.03% and it did not change with *Trichoderma* presence. However, in cultivar Gružanski zlatni content was 2.21 ± 0.18% and it decreased with *Trichoderma* treatment to 1.59 ± 0.08%. Content of phosphorus did not change significantly over the two cultivars and treatments; it ranged from the lowest 0.715 ± 0.045 ppm for Gružanski zlatni to the highest 0.79 ± 0.02 ppm for Narvik. Similarly, potassium content did not differ significantly either between treatments or between genotypes: it ranged from the lowest 2.78 ± 0.28 ppm in Gružanski zlatni to the highest 2.98 ± 0.14 ppm in Narvik (Table 4).

The reaction of two cultivars to the fungal treatment was the opposite in case of Mn. In cultivar Narvik it decreased, while in Gružanski zlatni it increased, but the changes were less than 10% (Table 4). The concentration of Fe and Cr were significantly higher in the *Trichoderma*-treated plants in comparison to the control in both cultivars. The changes of Fe and Cr were lower in Narvik, 23% and 59%, in comparison to Gružanski zlatni 72% and 100%, respectively. On the other hand, concentrations of Zn, Cu and Co were not affected by *Trichoderma* treatment and were similar in two cultivars as well.

Concentrations of Ni has decreased with the treatment in fruit of both cultivars, 20% in Narvik and 50% in Gružanski zlatni. However, concentrations of Pb responded significantly to the treatment only in Narvik, where it decreased by 50%.

BI for Mn, Ni and Cr ranged from 1.43 to 5.6. The lowest determined BI was for Fe, Pb and Co (˂1). However, high values of BI for Cu and Zn, ranging from 18.5–33, have been calculated. Values of BI in control conditions were similar in two cultivars. However, cultivar Gružanski zlatni had higher BI for Fe and Cr, and lower BI for Ni in response to *Trichoderma* treatment. Both cultivars responded to the treatment by lowering BI for Pb (Table 5).

### Principal Component Analysis

**Figure 2:**
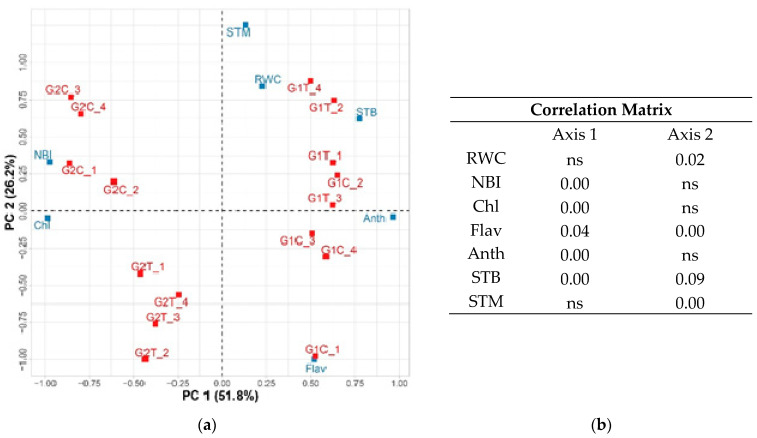
(**a**) PCA analysis of Narvik (G1) and Gružanski zlatni (G2) tomato cultivars, grown in the polytunnel without (C) or with *T. harzianum* application (T), involving chlorophyll (Chl), epidermal flavonols (Flav), nitrogen balance index (NBI), anthocyanin (Anth), relative water content (RWC (%)), stem diameter base (STB (cm)) and stem diameter middle (STM (cm)). (**b**) Correlation matrix (Pearson’s product correlation coefficient) with the associated *p* value between the principal components and the analyzed variables.

**Figure 3:**
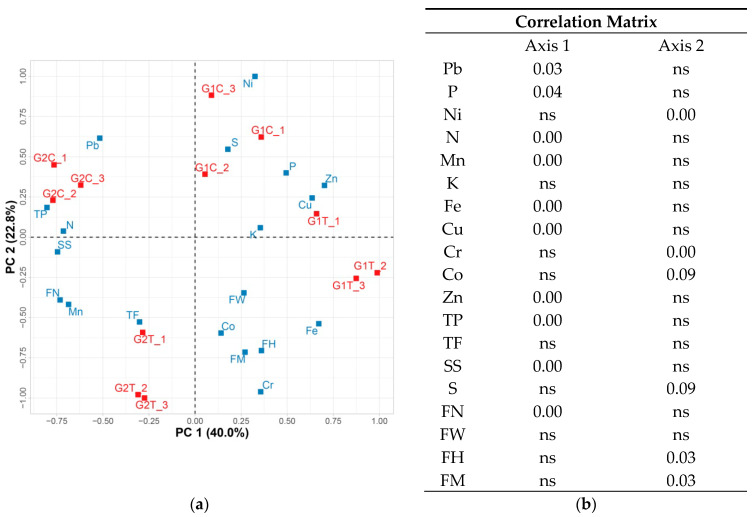
(**a**) PCA analysis of Narvik (G1) and Gružanski zlatni (G2) tomato cultivars, grown in the polytunnel without (C) or with *T. harzianum* application (T), involving: fruit weight (FW (cm 10^−2^)), fruit height (FH (cm 10^−2^)), number of fruit per plant (FN), fruit mass (FM (g)), total polyphenols (TP (GEA mg/100 gDW)), total flavonoids (TF (mg/100 gDW)), starch (S (mg/g DW)), contents of soluble sugars (SS (mg/g DW)), macroelements N (%), P (ppm), K (ppm) and heavy metals (ppm): Mn, Fe, Cu, Zn, Ni, Cr, Pb, and Co. (**b**) Correlation matrix (Pearson´s product correlation coefficient) with the associated *p* value between the principal components and the analyzed variables.

The effect of *Trichoderma* application on two tomato cultivars was visualized by performing PCA on parameters measured on plants (Figure 2) and fruits separately (Figure 3). The PCA of plant parameters, explained in total 78% of the variability (PC1 51.8%; PC2 26.2%). PC1 clearly separated two examined cultivars (Figure 2). Among the tested parameters, five out of seven were significantly correlated with the first axis. These parameters include NBI, Chl, Flav, Anth and STB. On the other hand, the RWC, Flav, STB and STM were significantly correlated with the second axis, which separated all treatments except one control sample of cultivar Narvik, which was in the group of samples from plants treated with *Trichoderma*.

The PCA of fruit parameters, explained in total 62.8% of the variability (PC1 40%; PC2 22.8%). Again, PC1 clearly separated two examined cultivars (Figure 3). Among the tested parameters, ten out of nineteen were significantly correlated with the first axis: contents of Pb, P, N, Mn, Fe, Cu, Zn, TP, SS, FN. Meanwhile, the second axis was determined by six out of nineteen parameters: contents of Ni, Cr, Co, S, FH, FM.

## 3. Discussion

It is known that some selected strains of *Trichoderma* promote plant growth and development, increase nutrient uptake and finally increase yields [24,25,26,27]. The efficient root colonization of this symbiont depends on the secreted effector molecules. About 20 *Trichoderma* effector proteins, belonging to three families (cerato platanins, hydrofobins and glycoside hydrolases) were analyzed so far [20]. The first two groups were the most examined in case of *T. harzianum*. One of the cerato platanin proteins that increases plant root colonization efficiency of *Trichoderma* is swolenin [21,28].

For this experiment we have selected strain *T.harzianum* SMZC 22660, due to high activities of *N*-acetyl-β-glucosaminidase, acid phosphatase and naphtol-AS-BI-phosphohydrolase. Acid phosphatase plays important role in solubilization of bound phosphates, making them available to plants [29]. *N*-acetyl-β-glucosaminidase is used as main ingredient of plant protection products isolated from *T. harzianum* P1 [30]. All three enzymes have also been considered important for intensive biocontrol of soil-borne pathogens because of their ability to degrade fungal cell walls [31]. Indeed we have observed good in vitro antagonistic activities of the *T.harzianum* SZMS22660 strain. For example, values of Biocontrol Index (BCI) calculated from the confronted cultures against *F. solani*, *R.solani*, *A. alternata*, and *P.cucurbitaceareum*, ranged from 70% to 100%, respectively (unpublished data).

Positive effects of *Trichoderma* on different tomato genotypes in terms of increasing fresh and dry root mass, shoot dry mass, and stem height were observed in most cases [32]. Beneficial plant growth effects following *T. harzianum* inoculation have often been explained by the improved plant nutritional status [33]. However, with some genotypes the treatment had no effect or was even detrimental [32]. In our experiments, performed on two tomato cultivars grown in polytunnel, no statistically significant differences of examined growth parameters, neither between cultivars, nor between *Trichoderma* treatments and controls were observed, which is not in accordance with our previous results [23].

The non-destructive measurements of leaf chlorophyll have been used in many plant species and different environmental conditions providing accurate estimate of leaf chlorophyll content [34]. In this paper, we have used Dualex 4 Scientific leaf-clip that shows a linear relationship with chlorophyll content [35]. This apparatus simultaneously measures epidermal flavonols on the same leaf area, based on the fluorescence excitation ratio method [36], comparing chlorophyll fluorescence induced by ultraviolet (UV) radiation (375 nm) with that induced by red light (650 nm) [37]. A positive relationship (*r*^2^ = 0.97) between measurements of total flavonoid content from laboratory analysis and amounts detected non-destructively has been confirmed in medicinal plants [38], grapevine [39] and white cabbage (*r*^2^ = 0.93) [40]. The content of chlorophylls and epidermal flavonols in the leaves of plants is known to be an important indicator of nitrogen status in a plant [41]. The chlorophyll content is positively correlated with the nitrogen content while content of epidermal flavonols is inversely correlated to nitrogen content [42]. In our experiment, the lower chlorophyll content and higher epidermal flavonol content in plants grown in the presence of *Trichoderma*, was statistically significant in Gružanski zlatni, and it was in correlation with decreased nitrogen content. Others have also observed reduction in chlorophyll content of plants inoculated with *Trichoderma asperellum* TaspHu1 strain [43] and *T. pseudoharzianum* T1 or *T. afroharzianum* T52, that was connected with downregulation of chlorophyll synthesis genes in the youngest leaves [44]. While there are reports where no significant increase of chlorophyll a and chlorophyll b in tomato leaves of plants treated with various *Trichoderma* strains [45], the increase content of photosynthetic pigments and photosynthetic capability in plants grown in presence of *Trichoderma* species was observed by others [15,16]. *Trichoderma* presence significantly affected epidermal flavonol content in the opposite directions in both tomato cultivars in our experiment. Others have shown that flavonoids can be induced by symbionts in roots and suggested their involvement in the regulation of a temporary defense response in the root triggered by the symbiont invasion [46]. The increase of total flavonoids and total polyphenols was also observed in leaves from 7 days up to 60 days after *Trichoderma* foliar application [14]. Moreover, six flavonoids that were significantly increased in bean plants in the presence of *Trichoderma* strain with major stimulation of plant growth have been identified [47]. In addition, the detailed metabolome and transcriptome analysis have shown that *T. harzianum* colonization strongly affects and remodels phenylpropanoid pathway of tomato plants [48]. Our data on decreased NBI in *Trichoderma* treated tomato plants of cultivar Gružanski zlatni indicates a shift from primary to secondary metabolism, and in accordance with previously cited papers, better resistance to diseases or insects might be expected [47,48].

Tomato fruit contains phenolic compounds among which flavonoids accumulate mainly in the peel and highly contribute to its antioxidant activity [49]. Some authors have observed the increase of total phenolic content in tomato fruit [50], grapes [27], edible onion part [51] and cucumber [52], while we observed the decrease of total phenolic compounds in both cultivars in *Trichoderma* treatment. Similar observations were reported in the study which evaluated the effects of three *Trichoderma* bioactive metabolites (BAMs) applied to strawberry plants [53]. They recorded reduced levels of total antioxidant capacity (TAC), total phenolic content (TPC), total and individual anthocyanins, as well as for antioxidant proteins, which appear to generally indicate a ROS-enriched environment in the BAM-treated fruits. As we have observed, the increase in total flavonoid content in fruit of *Trichoderma* treated cultivar Gružanski zlatni, the decrease of total phenolic content in fruit is probably connected with decreased content of non-flavonoids (phenolic acids) in this cultivar. On the other hand, in the study conducted on plum tomatoes in the greenhouse, it was observed that polyphenols in fruits were not affected by *T. harzianum* strain T22 [13]. The authors discussed that it might be due to the basal constitutive amount of polyphenols in plum tomatoes that is independent of influences by external stimuli. Variations in polyphenol and flavonoid content of tomato fruit depend on tomato fruit variety and this variation could be due to genetic differences as well as different environmental stress conditions and agricultural practices that affect the chemical composition of plants [54,55]. The different response of the flavonoid content in onion varieties to *T. asperellum* and T22 inoculation was also observed [51]. Ripening of tomato fruits is connected to sugar accumulation. The breakdown of starch is followed by increased content of hexose sugars during formation of red fruit, but depending on applied treatments the content of soluble sugars can decrease with the reduction of starch content during ripening [8]. Determination of sugar content is important for commercial market as it is directly connected with tomato fruit sweetness [56]. To our knowledge, in the literature there are no data showing the effect of *Trichoderma* on the starch and soluble sugar content in tomato fruit. According to our results, genotype Gružanski zlatni contains significantly more soluble sugars in comparison to Narvik, and this was not affected by *Trichoderma* treatment. However, *Trichoderma* addition decreased starch content in genotype Gružanski zlatni.

Our results on the content of micro-elements and heavy metals, in both soil and fruit, as well as the BI values, resemble values reported previously [6]. Though BI for examined elements was similar in two cultivars in control conditions, they responded differently to *Trichoderma* treatment. Cultivar Gružanski zlatni had a positive response of BI for Fe and Cr. The literature data on the effect of *Trichoderma* inoculation to the plant uptake of nutrients are not consistent. For example, some authors reported that in response to inoculation with *T. harzianum* concentrations of Cu, Fe, Mn and Zn significantly increased in roots, shoots and fruits of tomato plants [10] and cucumber roots [33]. This is probably connected with increased availability of the nutrients to plants, as shown for P and Fe in *T. asperellum*-treated cucumber plants [33]. In contrast, decreased accumulation of iron in leaves of tomato plants grown in presence of *T. harzianum* T34 was observed [57]. Moreover, decreased concentrations of Cu, Mn and Zn were observed in wheat plants grown on a calcareous medium and inoculated with *T. asperellum* [11]. Both authors suggest that the decreased concentrations of these elements in plants were due to the competition between plants and *Trichoderma*. Likewise, study on tomato plants grown in hydroponics, with specific nutrient deficiency, indicated that the effect of *T. harzianum* inoculation is depended on the deficient element. In case of either Fe- or Cu-deficiency, inoculation was accompanied with the increased uptake of these elements. However, in case of Zn deficiency, the uptake of Zn was suppressed in inoculated plants, due to competition with *Trichoderma* [58]. The decreased BI in response to *Trichoderma* treatment was observed for heavy metals: Ni in Gružanski zlatni and Pb in both cultivars. We have also observed reduced accumulation and translocation of toxic levels of elements in the presence of the same strain of *Trichoderma* previously [23]. Accumulation of Cu was decreased in onion plants in the presence of *T. asperellum* in conditions of toxic Cu concentrations [59]. Our results for decreased BI for Pb are in accordance with results of authors who observed decrease in uptake of Pb in four examined plant species *Miscanthus giganteus* L., *Salix* spp., *Phalaris arundinacea* L. and *Panicum virgatum* L. grown in *Trichoderma* presence [60].

Principal component analysis (PCA) is often used to distinguish significance of the relationship between many examined plant parameters. This multivariate analysis method aims to explain the correlation between a large set of variables in terms of a small number of underlying independent factors [61]. PCA confirmed that the main plant traits that were different in two cultivars in control conditions were the higher content of epidermal flavonols in leaves of Narvik, and higher NBI in leaves of Gružanski zlatni, as a consequence of higher chlorophyll and but lower epidermal flavonols content (Figure 2; Table 2). Gružanski zlatni reacted to *Trichoderma* treatment by decreased chlorophyll content, increased flavonol content and consequently decreased NBI (Figure 2; Table 2). However, treatment of cultivar Narvik was connected only with lower flavonol content (Figure 2). The main fruit traits that were different in two cultivars in control conditions were higher content of Ni in fruits of Narvik, and higher contents of TP and N, but lower content of Pb in Gružanski zlatni (Figure 3; Table 4) The important traits connected with *Trichoderma* treatment were the increased Fe and Cr in fruits of Narvik, and increased Mn and TF in fruits of Gružanski zlatni.

Although *T. harzianum* is one of the most accepted biopesticides, it remains unclear how the positive effects of this fungi depend on the plant genotype [32]. The same authors suggest that the response to *Trichoderma* spp. is under genetic control and it can be explained that some tomato genotypes have the ability to regulate colonization by *Trichoderma*. Different tomato genotypes in different ways perform a perception of effectors molecules to identify and enable colonization of the fungus [20]. In our experiment, better root colonization, determined by higher expression of swolenin gene in tomato roots of more responsive tomato cultivar was in correlation with observed positive effects of *Trichodrema*.

## 4. Materials and Methods

### 4.1. Plant Material and Growth Conditions

The field experiment was conducted in Selenča (45°24′52.1′′ N, 19°18′52.4′′ E), in the Province of Vojvodina, Serbia, on an organic vegetable farm in conditions that have been reported previously [62].

Shortly, tomato was grown in a 20 m length, 4.5 m width and 2.5 m maximal height polytunnel, without additional lighting or heating. There were two plots of the same size (10 × 4.5 m) and the experiment was conducted in a randomized block design with two replicates. Irrigation was done by the drip system, daily for one hour equally for all plots. Copper sulfate was only added as a pesticide at two times intervals, prior to flowering and prior to fruit formation. Two medium late tomato cultivars were grown in experiments. They are often used in production for industrial and market use, due to good fruit quality and resistance to diseases. Gružanski zlatni (G2) is resistant to *Fusarium oxysporum* f. sp. *Lycopersici, Verticillium albo-atrum* and it is also tolerant to Phytophthora infestans. Narvik (G1) is resistant to *Fusarium oxysporum* f. sp. *Lycopersici* and *Verticillium albo-atrum*. Seeds were sown in plug trays with a peat/perlite (3:1, *v/v*) medium in February, and after twenty days, the uniform seedlings were planted in plastic pots. At the beginning of April, 20 plants per treatment (about 20cm high) were transplanted into four rows (spacing 50 cm within the row and 50 cm between rows), within the same day. At the end of the June, ten uniform plants from each treatment were chosen to collect fully light-exposed, red-ripe fruits.

Ten uniform plants per plot were used for the experiments. Conidial suspension of the strain *Trichoderma harzianum* SZMC 20660 (8 × 10^6^ CFU/mL) was applied near the root zone, once during the growing period, in the first flowering phase.

Morphological and physiological parameters were measured 60 days after the treatment: soil water content (SWC), relative water content (RWC), epidermal flavonols (Flav) and total chlorophyll (Chl) (as described below), stem diameter base (STB), stem diameter middle (SDM), fruit weight (FW), fruit height (FH), fruit mass (FM) and number of fruit (FN) per plant. Four to seven red-ripe fruits [63] of similar size per plant were picked, measured (fruit width (FW) and height (FH)), washed, wiped, cut and stored at −80 °C for biochemical analysis.

### 4.2. Fungal Suspension Application

Strain *T. harzianum* SZMC 20660 (deposited in the Szeged Microbiological Collection (SZMC); Department of Microbiology) was isolated from the A horizon (5–30 cm) of agricultural soil used in organic agriculture. Species determination was based on their internal transcribed spacer (ITS) sequences [64].

Prior to preparation of fungal suspensions, *T. harzianum* isolate was pre-incubated at 25 °C in the dark. Suspensions were prepared as follows: pure culture of *T. harzianum* isolate was grabbed from a Petri dish, resuspended in 100 mL of tap water, and shaken for 2 h on 50 rpm. Conidial suspension (8 × 10^6^ CFU/mL) was applied near the root zone, once during the growing period, in the first flowering phase.

### 4.3. Detection of the Enzymatic Activity

*Trichoderma* strain SZMC22660 were preincubated on PDA medium at 25 °C for 5 days. Mycelia discs of 5 mm diameter were cut from the edge of petri dish and inoculated into 200 mL of sterile potato dextrose broth (PDB) liquid medium. Flasks were shaken for 7 days on 25 °C in the dark on an orbital shaker BIOSAN (Lithuania). Liquid culture was filtrated through sterile gauze and filtrate was used for the detection of extracellular enzymatic activity of 19 enzymes (alkaline phosphatase, esterase [C4], esterase-lipase [C8], lipase [C14], leucine arylamidase, valine arylamidase, cistine arylamidase, trypsin, α-chymotripsin, acid phosphatase, Naphtol-AS-BI-phosphohydrolase; α-galactosidase, β-galactosidase, β-glucuronidase, α-glucosidase, β-glucosidase, *N*-acetil-β-glucosamidase, α-mannosidase, and α-fucosidase) were determined by using the semi quantitative API ZYM system (bioMerieux, Marcy-l’Étoilem, France), according to the manufacturer protocol.

Sixty^-^five micro liters of each inoculum was transferred into each of the twenty API ZYM strip microtubes prefilled with 5 mL of distilled water for maintaining humidity and incubated at 37 °C in thermostat for 4 h. After incubation period a drop of ZYM A and ZYM B reagents was added to each of twenty wells.

The color reaction was read after 5 min according to the API ZYM reading color scale. According to this scale enzyme activities were scored as following: 0—no color, 1—low, 2—moderate and 3—high.

### 4.4. RNA Extraction and Expression Analysis of Swolenin by Real-Time RT-PCR

Six plants per treatment were harvested 60 days after the treatment and frozen in liquid N2. Root samples were harvested from lateral roots after quick wash of soil particles. Total RNA was extracted from ~100 mg of frozen and grinded samples by RNeasy plant mini kit, Qiagen (Hilden, Germany), according to the manufacturer’s procedure. cDNA was isolated from ~1 µg of total RNA using oligo(dT) as a primer, by RevertAid First cDNA Synthesis Kit, Fermentas (Waltham, MA, USA).

PCR conditions for the expression analysis of swolenin were the following: 1xBuffer, 3 mM MgCl_2_, 0.4 mM dNTP, 0.3 µM Primer, 1U Taq polymerase, 2,5 ng BSA. PCR programe was the following: 1′ 940C; 30 cycles 15′′ 940C; 30′′ 600C; 1′45′′ 720C; final extension 10′ 720C.

Swolenin expression was followed by amplification of a 121-bp fragment (Accession number EU370698; Forward primer GTGGCCAGTGTGGAGGTATT; Reverse Primer GTGAGGGATCGAGGTAGCTG). The β-actin was used as reference gene. It was followed by amplification of a 280-bp fragment (Accession number BT013524; Forward primer GACCTGCTCCAC-CATCTTCC; Reverse Primer CAGTGGAGTTGCCGACAAAG) [21].

### 4.5. Soil Sample Analysis

For chemical investigations, samples were air dried and sieved through a 0.2-mm sieve prior to analysis. Soil pH in water, humus content, free CaCO_3_ content, available phosphorus (P_2_O_5_), available potassium (K_2_O) and total N were done as described previously [17]. According to the content of CaCO_3_ (1.41 ± 0.01%), humus (2.9 ± 0.28%) and total N (0.15 ± 0.01%), the soil was weakly calcareous, moderate humic soil with optimal content of N. Available phosphorus content was high (29 ± 0.49 mg P_2_O_5_/100 g DW) and available potassium content (23 ± 1.48 mg K_2_O/100 g DW) was optimal. The soil was weakly alkaline (pH in H_2_O 7.9 ± 0.01).

### 4.6. Determination of Soil water Content (SWC) and Leaf Relative Water (RWC) Content

SWC was measured using Theta probe (Delta-T, Cambridge, UK) at a depth of 6 cm. RWC in leaves was determined from the ratio RWC = [(FW − DW)/(TW − DW)] × 100. FW, DW and TW were fresh, dry and turgid weights of 10 leaf discs (2 r = 1 cm), respectively.

### 4.7. Measurements of Epidermal Flavonols, Total Chlorophyll, Nitrogen Balance Index and Antocyanine

Indices of chlorophyll (Chl), epidermal flavonols (Flav) and ther ratio, NBI as well as antocyanine (Ant) were measured in vivo non-destructively with Dualex sensor (Force-A, Orsay, France) [65]. Measurements were done on ten uniform, fully developed and sun-exposed leaves of the same plants that were later used for measurements of plant and fruit morpho-physiological parameters.

### 4.8. Extraction of Phenolics and Soluble Sugars

The 500 mg of dried tomato samples (70 °C) were extracted two times with 5 mL of 80% methanol using ultrasonic bath for 30 min at room temperature. After sonication, the samples were centrifuged at 1000× *g* for 10 min. at 4 °C. The supernatants were collected and were evaporated to dryness by rotary evaporator (Heidolph, Laborota 4000, Schwabach, Germany) under reduced pressure at 40 °C. The residues after evaporation were dissolved in 50 mL of milliQ water and these solutions were used for analysis of total phenolics (TP), total flavonoids (TF) and soluble sugars (SS).

### 4.9. Determination of Total Phenolic Content (TP)

TP in the samples was determined using Folin–Ciocalteu’s reagent [66]. Tomato extracts were diluted 1:9 with distilled water. Briefly, an aliquot of diluted sample (70 µL) was mixed with 300 µL of Folin–Ciocalteu reagent (diluted 1:10). The samples were mixed and after 5 min 230 µL of 7.5% Na_2_CO_3_ was added, followed by incubation for 30 min at 45 °C in dark. After standing for 1 h at room temperature in dark, the absorbance was measured at 765 nm using a Shimadzu spectrophotometer (UV-1800, Shimadzu USA Manufacturing Inc., Canby, UR, USA).TP was expressed as milligrams of gallic acid equivalents per 100 g of dried sample (mg GAE/100 g).

### 4.10. Determination of Total Flavonoid Content (TF)

TF was determined using a colorimetric assay with aluminium chloride [67]. Briefly, an aliquot of sample (125 µL) was mixed with 625 µL of milliQ water and 37.5 µL of 5% NaNO_2_. After 6 min, 75 µL of 10% AlCl_3_ was added to form a flavonoid–aluminium complex. After 5 min 250 µL of 1 M NaOH and 138 µL of distilled water were added in the mixture to make the total volume of 1.25 mL. The absorbance was measured immediately against the blank at 510 nm. TFC was expressed as milligrams of quercetin equivalents per 100 g of dried sample (mg QE/100 g).

### 4.11. Determination of Soluble Sugars Content (SS)

SS content was determined by the anthrone colorimetric assay using glucose as the standard [68]. Briefly, 1ml of diluted dried tomato extract was added to 5 mL of anthrone reagent. After 5 min, the mixture was allowed to stand for 10 min at boiling water bath. The absorbance was read at 620 nm after 10 min of the sample cooling. SS was expressed as milligrams per g of dried sample.

### 4.12. Determination of Starch Content (S)

S content was determined according to the method described previously [69]. Briefly, the pellet after soluble sugars extraction mixed with 3.25 mL of 52% perchloric acid and 2.5 mL of distilled water. After 30 min at 0 °C, the mixture centrifuged at 17,000 g for 15 min. The supernatant was collected and the procedure was repeated. The obtained supernatant after repeated starch extraction was collected and mixed with the previous one. The final volume of supernatants was adjusted to 10 mL with distilled water after neutralisation to pH 7 with 4M NaOH and saturated solution of Na_2_CO_3_. The starch content in the obtained extracts was determined by anthrone colorimetric assay already described for determination of soluble sugar contents. The obtained results were multiplied with 0.9 (the glucose conversion factor to starch), [70] and were expressed as milligram per gram of dried sample.

### 4.13. Determination of Macroelements

Total nitrogen content was determined by Kjeldahl method, total phosphorus content and potassium content were determined by spectrophotometry and flame photometry, respectively, after dry destruction (ashing) at 550 °C [71].

### 4.14. Determination of Micronutrients and Heavy Metal Content in Soil and Tomato Fruit

Samples were dried at 70 °C and grinded to a fine powder, weighed sample, 1 g, was placed in a flask and treated with 3 mL of concentrated HNO_3_ for 4–5 h. A mixture of HNO_3_ and HClO_4_ (ratio of 2:1) was added (3 mL) and 33% H_2_O_2_. The mixture was heated at 120–130 °C for 5–6 h, until fumes stopped and resulting solution was clear. Then, 10 mL of deionized water was added into the flask and the solution was boiled again for 10–15 min until the volume was reduced to the half, cooled to room temperature and filtered using Whatman filter paper No. 42. The entire filtrate was mixed and made the volume up to 50 mL with deionized water. All digestions and measurements were performed in triplicate. Metal content was measured by atomic absorption spectroscopy (AAS) (Varian Spec-trAA 220 FS).

### 4.15. Bioaccumulation Index (BI)

The plant efficiency for uptake of elements in the soil and transfer to the edible part was estimated by the BI. It was calculated as the percentage of the concentration in the edible part of the plant to that in the cultivation soil [7].

$$BI=\frac{Metal\;concentration\;in\;fruit}{Metal\;concentration\;in\;soil}\;\times \;100$$

### 4.16. Statistical Analysis

Data are means of at least three replicates from four to ten different plants from each cultivar/treatment combinations. The results were analyzed by Tukey test using GraphPad Prism software. Principal component analysis (PCA) followed between groups analysis (BGA) was performed using the R platform (version 2.13.1, R Development Core Team, 2011) and the ade4TkGUI package was used [72]. The Pearson’s product moment correlation coefficient was calculated using the cor.test in order to disclose significant relationships between principal components and the variables analyzed (*p* < 0.05).

## 5. Conclusions

In our experiments, *Trichoderma* presence significantly affected Chl and Flav content and NBI in tomato leaves of cultivar Gružanski zlatni, which indicates a shift from primary to secondary metabolism. The increase in TF content in fruit of *Trichoderma*-treated plants of the same cultivar might contribute to its improved health promoting properties. The same cultivar responded to *Trichoderma* addition by decreased starch content, but increased BI for Fe and Cr and decreased BI for Ni. BI for Pb was decreased in both cultivars in response to *Trichoderma* treatment. Better root colonization, determined by higher expression of swolenin gene in tomato roots of more responsive tomato cultivar, was in correlation with observed positive effects of *Trichoderma*.

## Figures and Tables

**Table 1:** Extracellular enzyme activities as determined with API-ZYM system (alkaline phosphatase (A), esterase C4 (B), esterase lipase C8 (C), lipase C14 (D), leucine arylamidase (E), valine arylamidase (F), cysteine arylamidase (G), trypsin (H), α-chymotripsin (I), acid phosphatase (J), naphthol-AS-BI-phosphohydrolase (K), α-galactosidase (L), β-galactosidase (M), β-glucuronidase (N), α-glucosidase (O), β- glucosidase (P), *N*-acetyl-β-glucosaminidase (Q), α-mannosidase (R), α-mannosidase (S)). According to this scale, enzyme activities were scored as following: 0—no color, 1—low, 2—moderate and 3—high.

	A	B	C	D	E	F	G	H	I	J	K	L	M	N	O	P	Q	R	S
*T. harzianum* SZMC 22660	1	1	1	0	0	0	0	0	0	3	3	0	0	0	0	0	3	0	0

**Table 2:** Mean values of soil water content (SWC), relative leaf water content (RWC), indices of chlorophyll (Chl), epidermal flavonols (Flav), nitrogen balance index (NBI) and anthocyanin (Anth), as determined on ten plants of two tomato cultivars per treatment: cultivar Narvik (G1) and cultivar Gružanski zlatni (G2), without (C) or with *T. harzianum* application (T).

	SWC (%)	RWC (%)	Chl	Flav	NBI	Anth
G1C	24.50 ± 2.4 ^ab^	49.32 ± 3.7 ^a^	29.1 ± 1.8 ^c^	0.67 ± 0.03 ^a^	43.15 ± 1.2 ^c^	0.11 ± 0.005 ^a^
G1T	27.33 ± 3.0 ^a^	48.73 ± 4.7 ^a^	26.4 ± 1.2 ^c^	0.59 ± 0.02 ^b^	48.23 ± 3.8 ^c^	0.12 ± 0.008 ^a^
G2C	30.75 ± 0.8 ^a^	50.18 ± 4.3 ^a^	41.7 ± 1.1 ^a^	0.52 ± 0.03 ^c^	77.5 ± 1.5 ^a^	0.08 ± 0.008 ^b^
G2T	30.95 ± 1.1 ^a^	54.67 ± 2.6 ^a^	36.53 ± 1.2 ^b^	0.65 ± 0.03 ^ab^	61.25 ± 4.6 ^b^	0.09 ± 0.008 ^b^

Means with the same letter in the same column are not significantly different from each other according to Tukey’s test (*p* < 0.05). Error lines represent ± standard deviation of the mean. Different letters (^a, b, c^) indicate statistically significant differences according to Tukey’s test (*p* < 0.05).

**Table 3:** Mean values of plant (stem diameter base (STB) and stem diameter middle (SDM)) and fruit (fruit weight (FW), fruit height (FH), number of fruit (FN) and fruit mass (FM)) morphological characteristics, measured on ten plants of two tomato cultivars per treatment: cultivar Narvik (G1) and cultivar Gružanski zlatni (G2), without (C) or with *T. harzianum* application (T).

	STB (cm)	SDM (cm)	FW (cm × 10^−2^)	FH (cm × 10^−2^)	FN	FM (g)
G1C	16.36 ± 0.76 ^a^	10.6 ± 1.11 ^a^	60.03 ± 2.44 ^a^	57.8 ± 3.87 ^a^	12 ± 2 ^b^	130 ± 9.29 ^a^
G1T	16.7 ± 1.31 ^a^	12.36 ± 1.01 ^a^	61.96 ± 2.05 ^a^	61.3 ± 2.87 ^a^	11 ± 1 ^b^	143 ± 3.78 ^a^
G2C	13.87 ± 1.45 ^ab^	11.6 ± 1.05 ^a^	59.5 ± 4.37 ^a^	57.1 ± 1.42 ^a^	21 ± 3 ^a^	133 ± 4.57 ^a^
G2T	11.4 ± 0.87 ^b^	10.3 ± 0.81 ^a^	61.3 ± 3.61 ^a^	60.6 ± 1.57 ^a^	20 ± 1 ^a^	141 ± 11.35 ^a^

Means with the same letter in the same column are not significantly different from each other according to Tukey’s test (*p* < 0.05). Error lines represent ± standard deviation of the mean. Different letters (^a, b^) indicate statistically significant differences according to Tukey’s test (*p* < 0.05).

**Table 4:** Mean values of total polyphenols (TP), total flavonoids (TF), starch (S), contents of soluble sugars (SS), total nitrogen (N), phosphorus (P) and potassium (K) were measured on bulked samples of ten plants of two tomato cultivars per treatment: cultivar Narvik (G1) and cultivar Gružanski zlatni (G2), without (C) or with *T. harzianum* application (T).

	TP (GEA mg/100 gDW)	TF (mg/100 gDW)	S (mg/g DW)	SS (mg/g DW)	N (%)	P (ppm)	K (ppm)
G1C	1632.41 ± 68.74 ^b^	455.56 ± 47.13 ^b^	30.61 ± 3.81 ^ab^	225.18 ± 14.96 ^bc^	1.28 ± 0.03 ^c^	0.79 ± 0.02 ^a^	2.85 ± 0.28 ^a^
G1T	1408.29 ± 45.21 ^c^	333.26 ± 9.51 ^c^	37.65 ± 2.01 ^a^	192 ± 31.57 ^c^	1.27 ± 0.02 ^c^	0.77 ± 0.015 ^a^	2.98 ± 0.14 ^a^
G2C	2001.77 ± 52.61 ^a^	368.14 ± 29.02 ^c^	36.52 ± 4.62 ^a^	337.07 ± 34.51 ^a^	2.21 ± 0.18 ^a^	0.71 ± 0.045 ^a^	2.78 ± 0.28 ^a^
G2T	1710.63 ± 33.31 ^b^	548.41 ± 30.66 ^a^	22.74 ± 4.09 ^b^	284.09 ± 4.26 ^ab^	1.59 ± 0.08 ^b^	0.74 ± 0.035 ^a^	2.80 ± 0.00 ^a^

Means with the same letter in the same column are not significantly different from each other according to Tukey’s test (*p* < 0.05). Error lines represent ± standard deviation of the mean. Different letters (^a, b, c^) indicate statistically significant differences according to Tukey’s test (*p* < 0.05).

**Table 5:** Mean values of some micro-elements and heavy metals (mg kg^−1^), measured on fruits bulked from ten plants of two tomato cultivars per treatment: Narvik (G1) and Gružanski zlatni (G2), without (C) or with *T. harzianum* application (T). Concentrations of the same elements in the soil (b) and Bioaccumulation Index (BI) (c) are presented.

	Mn	Fe	Cu	Zn	Ni	Cr	Pb	Co
	Fruit (a)							
G1C	7.5 ± 0.1 ^c^	34.6 ± 1.0 ^b^	9.33 ± 0.15 ^a^	12.61 ± 0.61 ^a^	1.32 ± 0.05 ^a^	0.56 ± 0.005 ^b^	0.032 ± 0.01 ^a^	0.05 ± 0.01 ^a^
G1T	7.1 ± 0.05 ^d^	42.79 ± 0.11 ^a^	10.79 ± 1.55 ^ab^	12.79 ± 0.45 ^a^	1.08 ± 0.0 ^b^	0.89 ± 0.09 ^a^	0.0176 ± 0.03 ^b^	0.069 ± 0.02 ^a^
G2C	7.8 ± 0.1 ^b^	22.52 ± 1.00 ^c^	8.85 ± 0.02 ^ab^	11.16 ± 0.04 ^b^	0.995 ± 0.005 ^b^	0.48 ± 0.09 ^b^	0.030 ± 0.004 ^a^	0.05 ± 0.0005 ^a^
G2T	8.05 ± 0.1 ^a^	38.77 ± 2.81 ^a^	8.11 ± 0.36 ^b^	11.45 ± 0.79 ^ab^	0.49 ± 0.1 ^c^	0.99 ± 0.005 ^a^	0.0257 ± 0.045 ^a^	0.051 ± 0 ^a^
	Soil (b)							
	496.2 ± 3.9	13,685 ± 176.77	43.55 ± 1.21	39.13 ± 1.20	23.69 ± 0.59	23.07 ± 0.99	13.77 ± 0.94	9.043 ± 0.01
	BI (c)							
G1C	1.51	0.25	21.42	32.23	5.57	2.43	0.23	0.55
G1T	1.43	0.32	24.78	32.69	4.56	3.86	0.12	0.76
G2C	1.57	0.17	20.32	28.52	4.20	2.08	0.21	0.55
G2T	1.62	0.29	18.62	29.26	2.07	4.29	0.19	0.56

Means with the same letter in the same column are not significantly different from each other according to Tukey’s test (*p* < 0.05). Error lines represent ± standard deviation of the mean. Different letters (^a, b, c, d^) indicate statistically significant differences according to Tukey’s test (*p* < 0.05).

## Data Availability

The datasets used and/or analyzed during the current study are available from the corresponding authors on reasonable request.
